# Meteorological Register

**Published:** 1835-01-01

**Authors:** 


### METEOROLOGICAL REGISTER,

FROM SEPTEMBER 1 TO NOVEMBER 30.

By Messrs. HARRIS and Co., Mathematical Instrument Makers, 50, High Holborn.

| | Thermometer. | | Barometer. | | De Luc's Hygrometer. | | Winds. | | Atmospheric Variations. | | |
|---|---|---|---|---|---|---|---|---|---|---|---|
| 1834. | *max.* | *min.* | *9 a.m.* | *10 p.m.* | *9a.m.* | *10.pm.* | *9 a.m.* | *10 p.m.* | *9 a. m.* | *2 p.m.* | *10 p.m.* |
| Sept. 1 to 7 | 71 | 53 | 29.84 | 29.56 | 75 | 67 | SSW | SW | fine | cloudy | rain |
| 7 to 14 | 65 | 50 | 30.18 | .16 | 78 | 69 | S | SW | rain | fine | cloudy |
| 14 to 21 | 74 | 49 | .07 | .75 | 78 | 69 | E | NE | fine | cloudy | fine |
| 21 to 30 | 66 | 49 | .07 | .70 | 79 | 70 | NE SE | WSW v. | rain | cloudy | fine |
| Oct. 1 to 7 | 69 | 46 | .04 | .88 | 76 | 70 | SW | ESE | foggy | fine | foggy |
| 7 to 14 | 66 | 45 | 29.90 | .57 | 77 | 70 | SSW | SE | cloudy | fine | cloudy |
| 14 to 21 | 60 | 40 | 30.05 | .11 | 78 | 68 | WSW | SW | fine | fine | fine |
| 21 to 31 | 57 | 37 | .44 | .40 | 78 | 68 | NW | NE | rain | showery | fine |
| Nov. 1 to 7 | 65 | 46 | 29.92 | .25 | 80 | 74 | SSW | S | cloudy | fine | rain |
| 7 to 14 | 54 | 36 | 30.20 | .26 | 80 | 64 | N | NE | rain | rain | fine |
| 14 to 21 | 51 | 34 | .20 | .57 | 81 | 75 | NNW | E | fine | fine | rain |
| 21 to 30 | 52 | 35 | 29.90 | .06 | 84 | 76 | NNE | WSW | fine | fine | rain |

The quantity of Rain fallen in September, 1 inch.
— — — October, 36-100ths.
— — — November, 1 inch and 16-100ths.

